# Supplementary material for: Combined Magnetic Hyperthermia and Photothermia with Polyelectrolyte/Gold-Coated Magnetic Nanorods
Source: Polymers (Basel). 2022 Nov 14;14(22):4913. doi: 10.3390/polym14224913 (PMC9693010; doi:10.3390/polym14224913)
Supplement: Supplementary file 1 [file polymers-14-04913-s001.zip › polymers-1906948-supplementary.pdf]

SUPPLEMENTARY INFORMATION

Combined Magnetic Hyperthermia and Photothermia with Polyelectrolyte/Gold-Coated Magnetic Nanorods

Marina Lázaro [1], Pablo Lupiáñez [2], José L. Arias [3], María P. Carrasco-Jiménez [4], Ángel V. Delgado [5,*] and Guillermo R. Iglesias [6,*]

[1] Department of Applied Physics and Instituto de Investigación Biosanitaria ibs.GRANADA, NanoMag Laboratory, University of Granada, 18071 Granada, Spain
[2] Department of Applied Physics, NanoMag Laboratory University of Granada, 18071 Granada, Spain
[3] Department of Pharmacy and Pharmaceutical Technology, University of Granada, 18071 Granada, Spain
[4] Department of Applied Physics and Instituto de Investigación Biosanitaria ibs.GRANADA, NanoMag Laboratory, University of Granada, 18071 Granada, Spain
[5] Department of Applied Physics and Instituto de Investigación Biosanitaria ibs.GRANADA, NanoMag Laboratory, University of Granada, 18071 Granada, Spain;, NanoMag Laboratory University of Granada, 18071 Granada, Spain

* Correspondence: adelgado@ugr.es (Á.V.D.); iglesias@ugr.es (G.R.I.)

1. Geometrical characterization of the magnetic nanorods

To analyze the length and width of the nano-rods (Figure S1), a total of 100 nanoparticles were measured and their size was estimated from the microscopy images and JImage software. The value obtained for the length was 550 ± 90 nm (1:5 ratio).

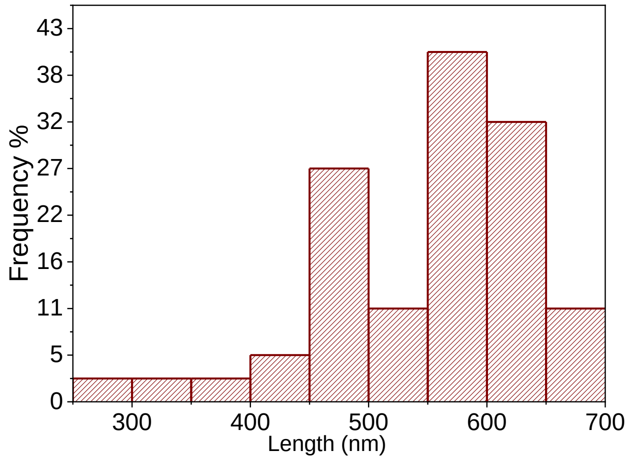

Figure S1. Histogram of magnetic nanorods length distribution.

2. Electrophoretic mobility of the MNRs

To check whether the different coating layers had been deposited, the electrophoretic mobility of the samples was measured (Figure S2). In the case of the PEI layer (cationic), the shift towards positive values of the mobility over the entire pH range due to a complete coating is observed. In the case of the gold seed coating (negatively charged) this effect is not so visible, evidencing that it is just a partial coating.

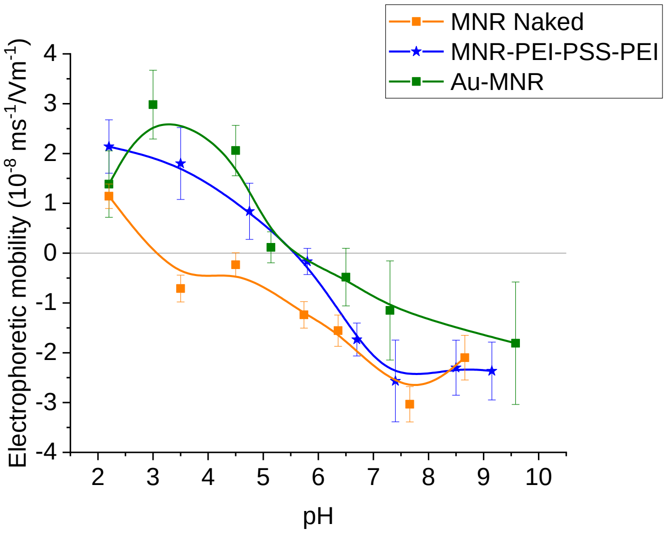

Figure S2. Electrophoretic mobility of MNR, coated with PEI-PSS-PEI and PEI-PSS-PEI-Au.

3. Optical absorbance

Figure S3 shows the optical absorbance spectra of pure magnetite and Au-coated nanorods.

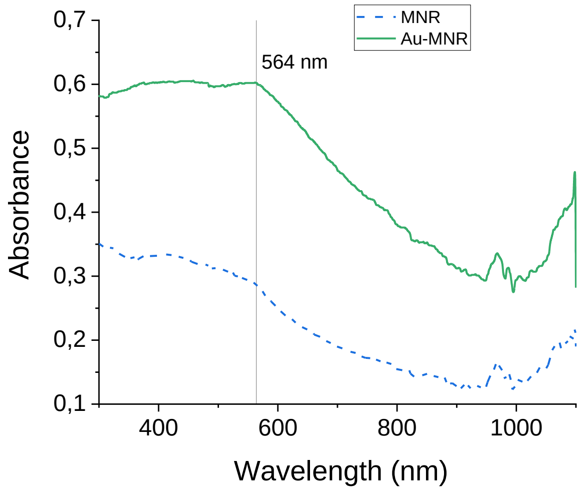

Figure S3. Optical absorbance of magnetite and gold coated nanoparticles.

4. Dual therapy

The first advantage in applying dual therapy is to achieve a decrease of the field strength and the irradiation power required to reach the therapeutic heating level. Figure S4

shows the temperature evolution when the two techniques were separately applied with the reduced intensities just mentioned. As observed, none of them could rise the sample temperature to the target value of 41-46 ºC, after reducing the field and laser powers to the values found optimal in dual application

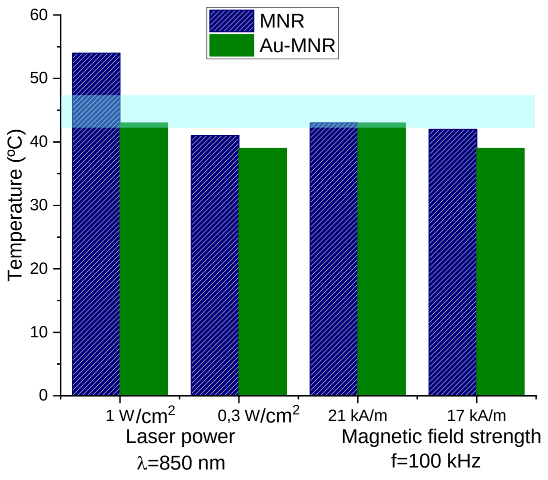

Figure S4: Maximum temperatures reached for magnetic hyperthermia and photothermia. Time of application 3 min. The blue band marks the desired temperature interval for hyperthermia.

5. Drug release

Figure S4 shows the experimental results of the accumulated drug release as a function of time without treatment, showing that even for times longer than those shown in the main text (Figure 14), the results obtained with hyperthermia, photothermia or dual therapy techniques are not achieved.

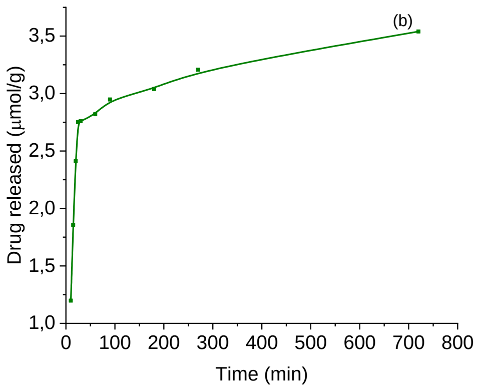

Figure S5. DOX release from magnetic nanoparticles in a pH 5 buffer and sink conditions. Extension of the time scale for the data obtained without hyperthermia treatment, as shown in Figure 14.
